# Changing phenotypic expression in a patient with a mitochondrial encephalopathy due to 13042G>A de novo mutation—a 5 year follow up

**DOI:** 10.1007/s11011-014-9645-x

**Published:** 2014-12-31

**Authors:** M. Schinwelski, B. Kierdaszuk, J. Dulski, K. Tońska, A. Kodroń, E. J. Sitek, E. Bartnik, A. Kamińska, H. Kwieciński, J. Sławek

**Affiliations:** 1 Department of Neurological and Psychiatric Nursing, Medical University of Gdansk, Gdansk, Poland; 2 Neurology Department, St. Adalbert Hospital, Copernicus Podmiot Leczniczy Sp. z o.o., Gdansk, Poland; 3 Department of Neurology, Medical University of Warsaw, Warsaw, Poland; 4 Institute of Genetics and Biotechnology, Faculty of Biology, University of Warsaw, Warsaw, Poland; 5 Institute of Biochemistry and Biophysics, Polish Academy of Sciences, Warsaw, Poland

**Keywords:** Encephalomyopathy, Mitochondrial diseases, Mitochondrial Encephalopathy with Lactic Acidosis and Strokelike episodes, Levetiracetam, Myoclonic Epilepsy with Ragged Red Fibres

## Abstract

Mutations in NADH dehydrogenase (ND) subunits of complex I lead to mitochondrial encephalomyopathies associated with various phenotypes. This report aims to present the patient’s clinical symptomatology in the context of a very rare 13042G>A de novo mutation and with an emphasis on changing phenotypic expression and pronounced, long-standing response to levetiracetam.

**Electronic supplementary material:**

The online version of this article (doi:10.1007/s11011-014-9645-x) contains supplementary material, which is available to authorized users.

Mutations in NADH dehydrogenase (ND) subunits of complex I lead to mitochondrial encephalomyopathies associated with various phenotypes (Lim et al. 2009). We have recently described a 13042G>A de novo mutation in a patient with an overlapping mitochondrial encephalopathy with lactic acidosis and strokelike episodes (MELAS) and myoclonic epilepsy with ragged red fibres (MERRF) clinical features. Genotyping for common mitochondrial mutations was negative, but clinical phenotype and the muscle biopsy suggested a mitochondrial disease. Thus, further genetic testing was performed and revealed a very rare 13042G>A mutation (Slawek et al. 2012). This report aims to present the patient’s clinical symptomatology in the context of a rare mutation and with an emphasis on pronounced and long-standing response to levetiracetam (LVT).

A 26-year-old Caucasian male, with unremarkable family history, was asymptomatic until the age of 18 when myoclonic jerks of the right upper extremity appeared followed by persistent headache, nausea and vomiting. At the age of 20 generalized (probably secondary) epileptic seizures occured. Valproate administration increased number of seizures and he was switched to oxcarbazepine (600 mg BID), which resulted in remission of seizures.

Neurological examination at the age of 21 showed myoclonic jerks of the right upper extremity at rest as well as with posturing. Myoclonic jerks were exacerbated with physical exercise and emotional stress and severely interfered with precise hand movements. After the next 10 months myoclonic jerks appeared proximally in the left upper extremity followed by ataxia, brisk tendon reflexes at the right side, writer’s cramp and startle reaction (see Video, Segment 1).

Laboratory studies performed at the age of 22 showed a normal serum lactic acid level of 9.45 mg% with an abnormal lactic acid curve during ischaemic muscle exercise. Creatine kinase activity was within the normal range. A 50 min video EEG disclosed a pattern characteristic for myoclonic epilepsy. Visual evoked potentials showed delayed P100 responses. Electromyography and nerve conduction studies were within normal ranges. Magnetic Resonance Imaging (MRI) showed multifocal hyperintensive lesions on T2 and FLAIR images in the right cerebral peduncle, close to the occipital horns of the lateral ventricles, in the left temporal lobe as well as in both frontal and parietal lobes (Fig. 1, segment a and b). Additionally, Magnetic Resonance Spectroscopy (MRS) indicated elevated lactate levels within the previously specified areas.

**Fig. 1:**
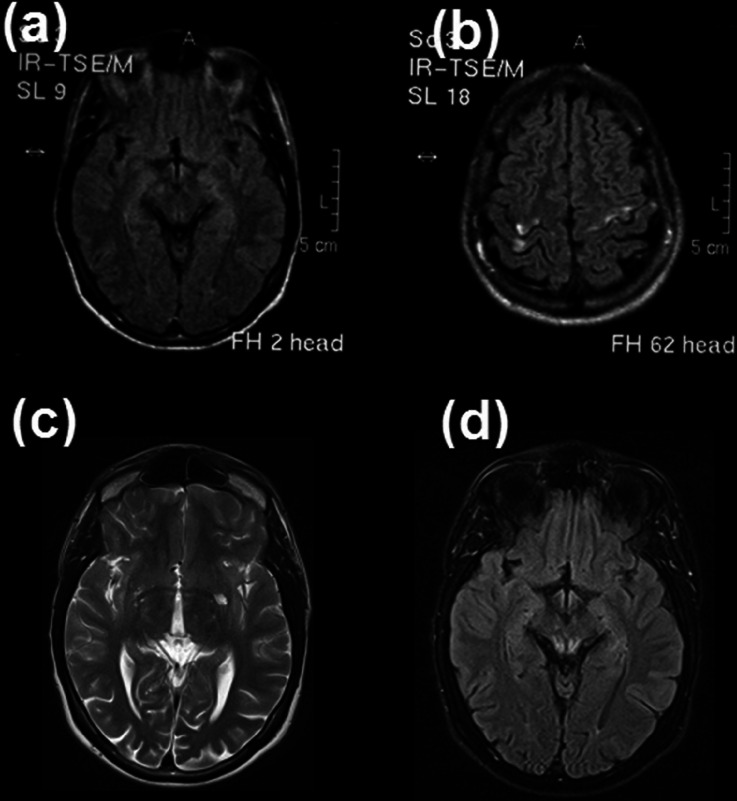
MRI T2 and Flair. Images (**a**) and (**b**) performed at the age of 22. (**a**) Hyperintensive lesion in right cerebral peduncle (**b**) hyperintensive cortical lesions in the frontal and parietal lobes bilaterally. Images (**c**) and (**d**) performed at the age of 26. (**c**) Hyperintensive lesion in left thalamus (**d**) symetrical dorsal hyperintensities of brainstem

The myoclonic jerks of upper limbs persisted despite the oxcarbazepine treatment. Therefore at the age of 22 the treatment was changed to levetiracetam 2 g daily with an excellent outcome—myoclonic movements significantly diminished (Unified Myoclonus Rating Scale score decreased from 69 to 18 points after 2 months). The patient’s quality of life improved dramatically and he was able to continue his education at the Technical University (see Video, Segment 2).

Improvement after LVT was seen over 36 months until other symptoms (beside myoclonic jerks) significantly handicapped our patient. At the age of 26 (5 years after the initial examination) he presented with generalized choreatic movements, exotropia in the right eye, vertical gaze limitation (especially downgaze), increased tendon reflexes on the left side, broad-based unsteady gait, disequilibrium and generalized ataxia. Ophtalmological examination revealed significant progression of optic nerve atrophy. A routine MRI scan showed new T2-weighted hyperintensities bilaterally in the thalamus and brainstem, which corresponded with the observed deterioration of the patient’s neurological status (Fig. 1, segment c, d) (see Video, Segment 3).

Mild cognitive dysfunction was stable at follow-up neuropsychological examinations at the age of 24 and 26. However, emotional deficits (leading to breakdown of relationships) and anosognosia progressed over time. The patient denied any psychological problems and failed to recognize the implications of motor symptoms (e.g. he continued to drive and engage in risky physical activity, unadjusted to his health state, ignoring medical recommendations).

During 5 years observation of the patient with de novo 13042 G>A mutation in subunit ND5 of mitochondrial complex I (CI) and overlapping symptomatology of MELAS, MERRF, PEO (progressive external ophthalmoplegia) and finally of LHON (Leber’s hereditary optic neuropathy) was noticed. Recently, there has been increased interest in the DNA gene encoding subunit 5 of complex I (MT-ND5), as a mutation hot spot for several overlap syndromes (Blok et al. 2007). Although13042G>A mutation was described earlier by Naini et al. (2005), we present a patient with a de novo mutation and long-term follow-up. This long-term observation shows the very complex pattern of neurological abnormalities evolving during 5 years from epilepsy, myoclonus and dystonia to choreatic movements with ophtalmoplegia and optic neuropathy.

In conclusion we suggest levetiracetam as a good symptomatic treatment of disabling myoclonus.

## Electronic supplementary material

Below is the link to the electronic supplementary material.

ESM 1 Video Segment 1: The patient at the age of 20–21. Video Segment 2: The patient at the age of 22, he has been on levetiracetam for 2 months. Video Segment 3: The patient at the age of 26. (WMV 18084 kb)
